# A placebo-controlled study of an herbal compound used for management of lower urinary tract disease in healthy cats

**DOI:** 10.3389/fvets.2026.1877164

**Published:** 2026-07-14

**Authors:** Cara E. Martin, Joseph W. Bartges, Cook F. English, Ashley P. Shaw, Sherry K. Cox

**Affiliations:** 1 Department of Small Animal Medicine and Surgery, College of Veterinary Medicine, The University of Georgia, Athens, GA, United States; 2 Department of Pathology, College of Veterinary Medicine, University of Tennessee, Knoxville, TN, United States

**Keywords:** calcium oxalate, feline, lower urinary tract signs, struvite, urolithiasis

## Abstract

**Introduction:**

Cystolithiasis is a common cause of feline lower urinary tract signs (LUTS). Urinary relative supersaturation (RSS) can be used to estimate an animal’s risk of crystallogenesis and urolithiasis, with high RSS values representing a potential increased risk of calculogenesis. Objectives were to determine whether an herbal supplement when compared with a placebo would decrease urinary excretion of electrolytes and minerals, increase urine volume, and decrease urine RSS for struvite (RSS S), calcium oxalate monohydrate (RSS COM), and calcium oxalate dihydrate (RSS COD).

**Methods:**

A randomized cross-over study in which 7 healthy male cats aged 10 months to 5 years received a herbal supplement or placebo for 2 weeks, followed by a five-day washout preceding the crossover treatment period. Voided 24-h urine samples were analyzed for pH and concentrations of sodium, potassium, chloride, calcium, phosphorus, magnesium, ammonia, oxalate, and citrate. RSS COM and RSS COD, and urine volumes were compared between supplement and placebo administration.

**Results:**

Six cats were included due to incomplete urine collection from one cat. Urine pH and analyte concentrations, 24-h urinary analyte excretions except phosphorus, which was decreased, and RSS COM and RSS COD did not differ between treatments. Struvite RSS was significantly lower in animals treated with the herbal supplement (*p* = 0.04).

**Discussion:**

Significantly lower RSS S with the herbal supplement suggests that it may be useful in preventing struvite crystallogenesis and calculogenesis in cats. This may be important for young to middle-aged cats (ages 1–7 years), who are at greatest risk for struvite crystal-associated lower urinary tract disease.

## Introduction

Cystolithiasis is a common cause of lower urinary tract signs (LUTS) in cats. Urolith and matrix-crystalline plug formation involve complex physiochemical processes. Major factors include: (1) urine supersaturation resulting in crystal formation (nucleation); (2) effect of inhibitors of mineral nucleation, crystal aggregation, and crystal growth; (3) crystalloid complexors; (4) effects of promoters of crystal aggregation and growth; (5) effects of non-crystalline matrix; and (6) and urine retention or slowed transit for the processes to occur (1–3). The most important driving force behind calculogenesis is urinary supersaturation with calculogenic substances; however, other factors are important. The goal of urinary crystal-related disease is to promote a reduced state of urinary saturation (4).

Degree of urinary saturation for different urinary crystals may be estimated by several methods, the most common is estimation of relative supersaturation (RSS). RSS is determined by measuring molar concentrations of analytes including ammonium, calcium, chloride, citrate, hydrogen (pH), magnesium, oxalate, phosphate, potassium, and sodium (and possibly cystine, sulfate, uric acid, and other compounds), in urine. These values are then entered into a computer program that iteratively determines the RSS for various crystals, such as struvite (RSS S), calcium oxalate monohydrate (RSS COM), and calcium oxalate dihydrate (RSS COD) (1, 5).

In cats presenting with LUTS, idiopathic cystitis (FIC) and urolithiasis account for over 80% of cases in cats less than 10 years of age (6). Incidence of lower urinary tract disease is currently unknown, but previous estimates in the United States and United Kingdom based on clinical signs have been reported to be 0.85 to 1.0% per year (7, 8). The proportional morbidity rate, defined as the frequency with which cases are seen at veterinary hospitals, has been reported to be 10% (4), although 1 to 6% is more commonly reported (9, 10). A more recent study evaluating 22,908 cats reported a proportional morbidity rate of 8% in cats with LUTS regardless of the cause. In this study 10% of cats were diagnosed with urolithiasis or urethral plugs; however, stone analysis was not performed. Cats between the ages of 2 and <7 were at an increased risk of obstruction secondary to urolithiasis or plugs (11). A study in Thailand evaluating 78 cats with LUTS reported struvite urolithiasis in 18% of cats (12) and a study evaluating 77 European cats with LUTS reported urolithiasis in 22% of cats (13).

Additionally, 80–90% of feline uroliths are composed of calcium oxalate or struvite (14–16). A study evaluating trends of stone composition in cats between 2005 and 2018 found that oxalate-containing uroliths decreased from 50.1% in 2005 to 37.7% in 2018. In contrast, the proportion of struvite-containing uroliths increased from 41.8% in 2005 to 54.5% in 2018 (17), which may shift initial treatment recommendations for non-obstructive uroliths regarding dissolution diets.

^1–341,5^Although several strategies for management of LUTS in cats have been investigated, a common recommendation is to promote dilution of urine, resulting in more frequent urination and bladder emptying, and to dilute crystallogenic constituents, initiators of urinary bladder pain, and potential initiators of inflammation (6). Larger volumes of urine typically increase rate of voiding frequency, reducing retention of calculogenic minerals. Feeding a canned diet is the most practical means of achieving increased water intake in cats and lowering calcium oxalate urine saturation and concentration of potential inflammatory mediators (6, 18); type of water bowl did not influence RSS for struvite or calcium oxalate (19). Higher dietary sodium intake increases water intake and urine volume in cats (20).

Sterile struvite uroliths form typically in cats between 1 and 10 years of age. Risk for struvite calculogenesis decreases after approximately 6–8 years of age in cats (21). They occur with equal frequency in male and female cats. Sterile struvite uroliths form because of alkaluria and increased concentrations of magnesium, ammonium, and phosphate, in the absence of bacteriuria with a concentrated urine, and innate risks for calculogenesis (22). Struvite is the most common mineral occurring in feline matrix-crystalline urethral plugs and crystallogenesis is similar to calculogenesis (23–25). Sterile struvite uroliths can be dissolved by feeding a diet that is magnesium-, phosphorus-, and protein-restricted, and that induces aciduria relative to maintenance adult cat foods (26, 27). This approach may also be used to prevent struvite calculogenesis (28).

Calcium oxalate uroliths form typically in cats older than 8 to 10 years of age (29). Calcium oxalate calculogenesis is dependent on urine oversaturation with calcium oxalate. Aciduria is associated with calcium oxalate calculogenesis (22). Hypercalciuria appears to be a major, but not the only, driving force for calcium oxalate calculogenesis in cats. Protocols for dissolving calcium oxalate uroliths are not available currently. Feeding diets formulated to decrease urinary calcium oxalate RSS may be beneficial; however, no long-term studies have been performed to validate this (22, 30).

In addition to conventional therapy using modified diets, traditional Chinese and Western herbs have been recommended. Choreito, a traditional Chinese medicinal herb, decreased the risk of struvite calculogenesis in adult cats; however, no benefit was found in another study of three commonly used herbal treatments, San Ren Tang, Wei Ling Tang, and Alisma (31–34). In a study evaluating Choreito, no differences were found in 24 h urinary analyte excretions, urine volume, urine pH or urinary saturation for calcium oxalate or struvite between treatments and compared to placebo (35).

Objectives of this study were to determine whether a multi-herbal supplement^1^ would be associated with differences in urinary excretion of analytes, increased urine volume, and decreased urine RSS S, RSS COM, and RSS COD when compared with a placebo. The multi-herbal supplement is recommended for use in cats with lower urinary tract disease and is purported to have anti-inflammatory, antioxidant, and analgesic effects. Theoretically, this supplement may be used as a prophylaxis in cats without concurrent lower urinary tract disease. The herbal supplement contains a mix of herbs and is a flavorless liquid that is reportedly safe to be administered alone or in combination with other therapies used to support bladder and kidney health. There are no prospective studies evaluating the efficacy of this herbal supplement. We hypothesized that administration of the multi-herbal supplement would be associated with differences in urinary analyte excretion, increased urine volume, and decreased urine saturation for calcium oxalate monohydrate, calcium oxalate dihydrate, and struvite when compared with placebo.

## Materials and methods

Seven healthy, adult castrated male cats aged 10 months to 5 years were evaluated in a double-masked, randomized, cross-over design study. Cats were determined to be healthy based on results of physical examination and laboratory evaluation including CBC, biochemical analysis, urinalysis, and aerobic microbial culture of the urine collected by cystocentesis. Cats were randomized to one of two treatments using a random numbers generator, a placebo or treatment (supplement). Cats received supplement or placebo at 1 mL/cat every 12 h. The supplement is commercially available and contains the following (per 1 mL): 33 mg stoneroot, 33 mg parsley piert, 25 mg hydrangea, 25 mg gravel root, 17 mg *Echinacea purpurea*, 17 mg marshmallow, and 17 mg of Oregon grape. The placebo was 1 mL/cat of inert ingredients used in the product (purified water and glycerin) by mouth every 12 h. The initial treatment period lasted 2 weeks, followed by a five-day washout preceding the crossover treatment period such that all cats received placebo or supplement during the study.

Determination of urinary saturation requires a timed urine sample; therefore, cats were housed in individual cages equipped with a modified litter box that permitted separation of feces from urine. Prior to urine collection, the urinary bladder was palpated and if urine was still present after voiding, cystocentesis was performed using a 1-inch or 1 ½-inch 22G hypodermic needle and a 6 to 20 mL syringe depending on the size of the cat and the size of the urinary bladder to remove remaining urine. All urine voided by the cat over the next 48 h was collected in sealed containers containing thymol to minimize bacterial contamination. Containers were emptied into a larger collection container and refrigerated during the collection period. At the end of the 48-h period, the urinary bladder was palpated and if urine was present, a cystocentesis was again performed to remove urine. Urine samples were analyzed for sodium, potassium, chloride, calcium, phosphorous, magnesium, and creatinine using an automated chemistry analyzer,^2^ citrate and oxalate by ion chromatography, pH by pH electrode, and ammonia by ion-select electrode.

Cats were fed a dry-formulated feline maintenance adult diet^3^ and water was always available. Cats were allowed to group play and intermingle and received daily handling but were individually housed to acclimate to the modified litter pan for timed urine collections.

Twenty-four-hour urinary excretion of various analytes were calculated by converting data to a 24-h period (amount per kilogram of body weight per 24 h) and urinary saturation was estimated using a computer program^4^ (36). Shapiro–Wilk test was used to analyze data for normal distribution. With normally distributed data, a two-tailed, paired t-test was used to assess differences in urinary electrolyte and mineral excretion, urinary saturation (RSS S, RSS COM, and RSS COD), and urine volume between supplement and control groups. A Wilcoxon signed-rank test was performed if data lacked normal distribution. Statistical analysis was performed using microcomputer software program.^5^ RSS values and urine volumes were compared with one-tailed t-tests. All procedures involving animals were reviewed and approved by the Institutional Animal Care and Use Committee of the University of Georgia Animal Welfare Assurance (IACUC protocol number: A2017 03-030-Y1-A0).

## Results

**Figure 1:**
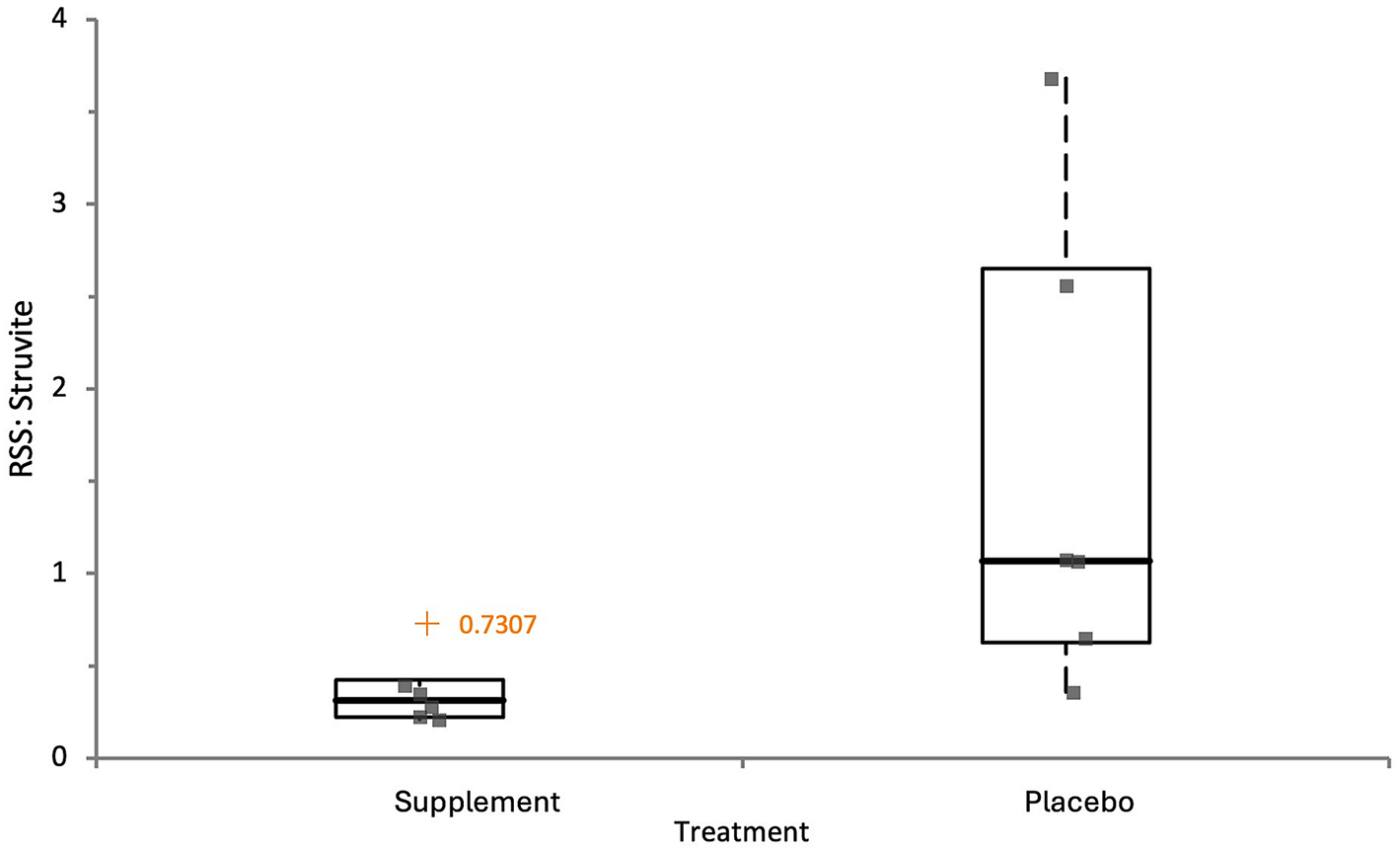
Comparison of struvite relative supersaturation values between supplement and placebo (*p* = 0.04) in healthy cats.

One cat, 5 years of age, was excluded from analysis due to incomplete urine collection. Body weight, urine pH, urine volume, urine analyte concentrations and 24-h analyte excretions except phosphorus, which was decreased, and RSS COM and RSS COD were not significantly different between cats receiving supplement compared with placebo (Table 1). Mean RSS S was significantly lower when cats received supplement (supplement = 0.36 ± 0.19, placebo = 1.57 ± 1.28, *p* = 0.04; Figure 1).

**Table 1:** Results of body weight, 24-h parameters and relative supersaturation for struvite (RSS_MAP_) and calcium oxalate (RSS_CO_) for 6 male domestic short-haired cats receiving herbal supplement or placebo.

Variable	Units	Supplement	Placebo	*p*-value
Body weight	Kg	5.30 +/− 0.72	5.13 +/− 0.95	0.43
Urine volume	L/24 h	0.081 +/− 0.029	0.096 +/− 0.028	0.37
	mL/kg/24 h	15.86 +/− 6.93	18.70 +/− 1.77	0.81
pH		6.45 +/− 0.25	6.99 +/−0.67	0.14
Sodium	M/L	0.123 +/− 0.019	0.125 +/− 0.019	0.82
	mEq/kg/24 h	1.85 +/− 0.58	2.28 +/− 0.33	0.24
Potassium	M/L	0.120 +/− 0.021	0.105 +/− 0.013	0.06
	mEq/kg/24 h	1.77 +/− 0.49	1.94 +/− 0.31	0.58
Calcium	mM/L	0.370 +/− 0.053	0.324 +/− 0.097	0.30
	mg/kg/24 h	0.23 +/− 0.09	0.24 +/− 0.08	0.85
Magnesium	mM/L	1.001 +/− 0.697	0.650 +/− 0.640	0.43
	mg/kg/24 h	0.384 +/− 0.314	0.294 +/− 0.297	0.69
Ammonia	mM/L	81.89 +/− 29.85	105.3 +/− 27.61	0.18
	mM/kg/24 h	1.239 +/− 0.546	2.008 +/− 0.801	0.12
Phosphorous	M/L	0.032 +/− 0.006	0.029 +/− 0.004	0.17
	mg/kg/24 h	44.89 (24.4, 61.3)	50.75 (41.4, 63.5)	0.04
Oxalate	mM/L	1.067 +/− 0.144	0.974 +/− 0.096	0.15
	nM/kg/24 h	16.29 +/− 5.37	17.92 +/− 2.70	0.52
Citrate	mM/L	0.801 +/− 0.442	0.785 +/− 0.498	0.90
	uM/kg/24 h	11.53 +/− 5.89	14.05 +/− 8.16	0.49
Chloride	M/L	0.129 +/− 0.029	0.133 +/− 0.025	0.73
	mEq/kg24h	1.88 +/− 0.45	2.41 +/− 0.34	0.13
Creatinine	mg/kg/24 h	41.9 +/− 10.7	40.7 +/− 3.2	0.83
RSS struvite		0.364 +/− 0.193	1.565 +/− 1.284	0.04
RSS calcium oxalate dehydrate		0.942 +/− 0.217	0.518 +/− 0.467	0.92

Data are represented as mean +/− standard deviation if normally distributed or as a median (minimum, maximum) if not normally distributed.

## Discussion

Calculogenesis is a complex reaction driven by urine saturation, a combination of urine metabolite concentrations and inhibitor concentrations (1). Thus, an RSS value provides an estimate for the degree of urinary saturation for that given compound. RSS S is especially important in young adult cats who are at greater risk for struvite stones and struvite crystal – matrix plugs than older cats. Our study found that the supplement significantly decreased 24-h excretion of phosphorus and RSS S. Lower RSS S values may be the result of decreased urine phosphorus, a combination of changes between Mg, NH4, and PO4 excretion values, increased concentrations of inhibitory compounds, or decreased urine pH associated with increased struvite solubility. Lack of a significant difference in RSS COM and RSS COD may be due to the small sample size and the age range of our cats, as urinary saturation for calcium oxalate in cats increases with age (29, 37–40).

Significantly lower RSS S values with the supplement suggest that one or more ingredients may be useful to prevent struvite crystallogenesis and calculogenesis as well as urethral plug formation in cats. Recent global data reported by the Minnesota Urolith Center stated the mineral composition of feline urethral plugs was 99% struvite (41). Therefore, this therapy is especially important for cats aged 1 to 7 years, which are at greatest risk for struvite calculogenesis (15, 25) and cats with matrix-crystalline urethral plugs (41). These diseases are associated with struvite calculogenesis when compared to other stone types in cats (25). We did not find a significant difference in urine volume between cats receiving the supplement and those receiving a placebo, suggesting that significantly increasing urine volume is not as important as lowering RSS values. Use of the supplement may be beneficial in cats with recurring struvite related diseases as it decreases urinary saturation with struvite. Continued investigation of this supplement in cats predisposed to developing struvite uroliths is warranted.

RSS COM and RSS COD were not lower when cats received the supplement when compared with placebo. While this may reflect lack of efficacy of the supplement on urinary calcium oxalate saturation, it may also be due to using young adult male cats as calcium oxalate urolithiasis tends to occur in middle-aged and older cats (29, 37–40). Additionally, urine pH was more acidic when cats received supplement than placebo and RSS COM and RSS COD are higher when urine is more acidic (42). Whether this effect would be present in older cats is unknown.

There were limitations to this study. Although a single diet was fed, the study period was short. Over longer periods of time, diet can contribute to changes in pH which may predispose to crystallogenesis; however, if calculogenesis can be prevented with this supplement, diet change may not be necessary in all patients. Another limitation of this study was the small sample size, which may have precluded the ability to detect significant differences in RSS COM and RSS COD or other urine metabolites. Regardless, significant differences in RSS for struvite were observed.

The population of cats in this study were young adults. Struvite urolithiasis tends to occur more commonly in younger cats whereas calcium oxalate urolithiasis is more prevalent in older cats. It is possible that a significant difference in RSS COM and RSS COD may have been found if a larger population of cats with a more comprehensive age range was evaluated. Additionally, the small number of patients were healthy, non-urolith forming cats and urine collection may not have been complete despite the use of modified litter boxes.

## Conclusion

There was a significant decrease in struvite saturation even with the previously mentioned limitations suggesting determination of relative supersaturation is a better determinant of risk of calculogenesis than electrolyte and mineral concentrations or excretions. This supplement may be beneficial for managing struvite-associated urinary disease in cats; however, further investigation in clinical cases is still needed to evaluate efficacy in cats with a predisposition to development of urolithiasis. While the supplement is reportedly safe for long-term use, extended safety studies are needed.

## Data Availability

The original contributions presented in the study are included in the article/supplementary material, further inquiries can be directed to the corresponding authors.

## Footnotes

1 Tripsy for Cats. Lot number 33795. NHV Natural Pet Products, Vancouver, BC, Canada.

2 Abaxis Chemistry Analyzer, University of Tennessee College of Veterinary Medicine Clinical Pathology Laboratory, Knoxville, TN.

3 Feline Maintenance; SportMix, Midwestern Pet Foods, Evansville, IN.

4 EQUIL 93b, University of Florida, College of Medicine, Gainesville, FL.

5 Analyze-It Software, v. 4.81.6, Leeds, United Kingdom.
